# Post-refractive Surgery Dry Eye: A Systematic Review Exploring Pathophysiology, Risk Factors, and Novel Management Strategies

**DOI:** 10.7759/cureus.61004

**Published:** 2024-05-24

**Authors:** Saif K Dossari

**Affiliations:** 1 Department of Surgery, King Faisal University, Al-Hofuf, SAU

**Keywords:** neurotrophic keratitis, inflammation, ocular allergies, refractive surgery, dry eye disease, anterior eye therapeutics

## Abstract

Dry eye disease frequently manifests following corneal refractive procedures, significantly impacting patients’ quality of life. This review systematically synthesizes current evidence on the pathophysiological mechanisms, risk factors, and therapeutic interventions for post-refractive surgery dry eye. Following the Preferred Reporting Items for Systematic reviews and Meta-Analyses (PRISMA) guidelines, a systematic review of literature published until August 2023 was conducted, focusing on post-refractive surgery dry eye. Eighteen relevant studies were identified through screening and eligibility assessment. A qualitative synthesis of outcomes was performed using narrative and thematic analysis methods. Surgically induced neurotrophic deficiency, stemming from nerve transection, triggers a cascade of events including apoptosis, inflammation, and lacrimal dysfunction, ultimately leading to tear film instability. Risk factors such as female gender, thyroid eye disease, meibomian gland dysfunction, higher ablation depths, and the use of LASIK over surface ablation exacerbate the condition. While conventional treatments like artificial tears provide temporary relief, emerging interventions such as nerve growth factors, matrix metalloproteinase inhibitors, serum eye drops, and specialized contact lenses show promise in promoting nerve regeneration and epithelial healing. Strategies such as customized ablation profiles, smaller optical zones, and nerve-sparing techniques like small incision lenticule extraction demonstrate potential advantages. A multifaceted therapeutic approach targeting neuroprotection, anti-inflammatory mechanisms, and tear film stabilization is imperative for effectively managing post-refractive surgery dry eye. Future research should focus on evaluating prognostic biomarkers, exploring precision medicine approaches, and investigating neuroprotective adjuvants to further enhance treatment outcomes.

## Introduction and background

Dry eye disease is a multifactorial condition affecting the ocular surface and tear film, characterized by symptoms of ocular discomfort, visual disturbances, tear film instability, and potential damage to the ocular surface [1]. It has emerged as one of the most common complications following refractive surgery procedures such as laser-assisted in situ keratomileusis (LASIK) and photorefractive keratectomy (PRK) [2]. Post-refractive surgery, dry eye can significantly impact patients’ visual outcomes and quality of life in the long term [3].

In recent years, there has been growing research into understanding the complex pathophysiological mechanisms, risk factors, and therapeutic approaches for managing post-refractive surgery dry eye [4-6]. This review summarizes the current evidence on the pathophysiological changes to the ocular surface and lacrimal functional unit that contribute to dry eye after corneal refractive procedures. Key preoperative risk factors that predict the development of post-refractive surgery dry eye are highlighted. Finally, an overview is provided of the various management strategies described in the literature for alleviating signs and symptoms in these patients.

Pathophysiology

The pathophysiology of post-refractive surgery dry eye is multifactorial, involving complex alterations to neural pathways, tear film, and the ocular surface [7]. Corneal refractive procedures induce changes to corneal innervations through the severing of afferent sensory nerves in the anterior stroma [8,9]. This neurotrophic epitheliopathy impedes corneal wound healing and triggers inflammation, impairing the stability of the ocular surface and tear film [10].

Surgical disruption and abnormal regeneration of sub-basal nerves lead to reduced corneal sensitivity in the early postoperative period [11,12]. Patients experience significantly lowered sensitivity to mechanical, chemical, and thermal stimulation of the cornea after LASIK and PRK [5]. This corneal hypoesthesia likely impairs lacrimal functional unit homeostasis through diminished trigeminal reflex arcs, causing diminished tear production and instability of the tear film after surgery [13].

Surgical manipulation during refractive procedures also directly damages the corneal epithelial stem cells present in the limbus [14]. Loss of limbal stem cells can impair epithelial cell renewal and healing postoperatively [15]. Histological analyses show significant changes to epithelial and stromal structures after LASIK, including epithelial thinning, apoptosis, activation of stromal keratocytes, and disruption of anchoring complexes at the epithelium-basement membrane interface [16]. These ultrastructural changes destabilize corneal and conjunctival epithelia after surgery [17].

Together, the neural and structural changes involving corneal denervation, epithelial damage, and inflammation initiate lacrimal functional unit dysfunction, characterized by alterations in tear composition, volume, and clearance [18]. These ultimately culminate in the signs and symptoms characteristic of dry eye disease after refractive surgery [19].

Risk factors

Multiple systemic and ocular factors have been studied as potential preoperative risk factors to identify patients at higher risk of developing post-refractive surgery dry eye [3]. Key risk factors consistently reported in large retrospective analyses and systematic reviews include female sex, the presence of preoperative dry eye signs and symptoms, LASIK (compared to surface ablation techniques), intraoperative complications causing extensive corneal flap damage, and higher ablation depths and optical zones [20,21].

Females demonstrate accelerated epithelial apoptosis and nerve damage after surgery compared to males [22]. Hormonal variations in estrogen and progesterone levels are postulated to impact wound healing responses [23]. Preexisting dry eye with signs of meibomian gland dysfunction, tear film instability, and chronic inflammation predisposes patients to further exacerbations after corneal surgeries [24]. Higher ablation depths directly correlate with greater loss of anterior stromal keratocytes and nerve damage [25]. Thus, these factors contribute to the neurotrophic epitheliopathy that amplifies dry eye after refractive procedures [26].

Various questionnaires have been designed to screen patients preoperatively, such as the Standard Patient Evaluation of Eye Dryness (SPEED) questionnaire [27-30]. However, more studies are required to determine optimal predictive cutoff values to identify surgically amenable candidates versus poor candidates requiring optimization of ocular surface disease prior to corneal refractive surgery [31].

Management approaches

A wide range of therapeutic approaches have been explored for managing post-refractive surgery dry eye, targeting the various pathways underpinning its multifactorial pathophysiology [32]. These include conventional dry eye treatments such as ocular lubricants, lid hygiene, anti-inflammatory agents, and punctal occlusion [10]. Novel approaches under investigation also show promising results, such as autologous serum eye drops, nerve growth factors (NGFs), matrix metalloproteinase inhibitors (MMPIs), Rose Bengal dye phototherapy, and scleral lens therapy [33].

Artificial tear supplements provide transient lubrication and protect the ocular surface from desiccation [34]. Preservative-free lubricants are preferred to limit toxicity from prolonged use after refractive procedures [35]. Lid hygiene and warm compresses help restore meibomian gland function altered by corneal denervation [36]. Topical corticosteroids (loteprednol and difluprednate) and immunomodulatory agents (cyclosporine and lifitegrast) reduce inflammation in the lacrimal glands and ocular surface [37]. While effective for symptomatic relief, these anti-inflammatory agents come with side effects that limit their long-term use [38].

More targeted approaches in development include NGF mimetics and MMPIs. NGFs directly promote corneal epithelial healing and nerve regeneration [39]. MMPIs may improve epithelial basement membrane integrity and reduce activation of nociceptive pathways mediating ocular pain after corneal surgery [40]. Other novel techniques, like autologous serum eye drops, provide essential tear components such as vitamin A, fibronectin, and epidermal growth factors to stimulate epithelial healing [41]. Phototherapeutic modification of ocular surface tissues using Rose Bengal dye also shows promise in preliminary trials [42].

Significant progress has been made in unraveling the complex pathophysiology of dry eye disease secondary to corneal refractive surgeries [3]. Damage to corneal nerves and ocular surface stem cells after LASIK/PRK initiates dysfunction of lacrimal functional unit homeostasis, impairing tear film stability, composition, and clearance [43]. Multiple preoperative risk factors relating to patient demographics, corneal characteristics, and surgical techniques predispose to postoperative dry eye [44]. Conventional dry eye treatments provide symptomatic relief, but more targeted approaches are upcoming, targeting nerve regeneration, epithelial healing, and anti-inflammation [45]. Further studies should explore if modulation of surgical techniques and optimized perioperative management can improve outcomes. This review summarizes the current evidence on the pathophysiological changes to the ocular surface and lacrimal functional unit that contribute to dry eye after corneal refractive procedures.

## Review

Materials and methods

Search Strategy and Selection Criteria

The foundation of this systematic review was built upon a meticulous adherence to the guidelines set forth by the Preferred Reporting Items for Systematic reviews and Meta-Analyses (PRISMA) guidelines [46], ensuring a stringent commitment to transparency and comprehensiveness. Following the methodology outlined in the PRISMA Protocols (PRISMA-P) statement [47], a meticulously crafted research protocol was developed and subsequently registered with PROSPERO (CRD42023478362), ensuring the systematic and methodical execution of the review process.

To ensure a thorough and exhaustive exploration of available literature, an extensive search strategy was meticulously devised. Esteemed databases such as Embase, Medline ALL (Ovid), Web of Science Core Collection, Cochrane Central Register of Controlled Trials (Wiley), and Google Scholar were diligently explored. The most recent search, conducted on September 17, 2023, ensured the inclusion of the most current and relevant research findings. The search strategy was intricately designed, incorporating a refined amalgamation of medical subject headings (MeSH) and carefully selected keywords pertinent to post-refractive surgery dry eye (Table 1). Encompassing the multifaceted aspects of the condition, including its pathophysiology, associated risk factors, and various management approaches, the strategy aimed for comprehensive coverage of the topic.

**Table 1:** A comprehensive overview of studies focusing on the pathophysiology, interventions, and management approaches related to DES across diverse clinical setting BCVA, best corrected visual acuity; CDVA, corrected distance visual acuity; CFS, corneal fluorescein staining; CFT, central foveal thickness; CsA, cyclosporine A; DES, dry eye syndrome; E-PRP, eye platelet rich plasma; FS-LASIK, femtosecond laser-assisted in situ keratomileusis; HOA, higher-order aberration; LASIK, laser-assisted in situ keratomileusis; MMP, matrix metalloproteinase inhibitor; NIBUT, noninvasive tear break-up time; NSAIDs, non-steroidal anti-inflammatory drugs; OSI, Objective Scatter Index; OSDI, Ocular Surface Disease Index; PRK, photorefractive keratectomy; SIT, Schirmer I test; SPK, superficial punctate keratopathy; TBUT, tear breakup time; TFO, tear film osmolarity; UDVA, uncorrected distance visual acuity

Study	Study design	Intervention	Control	Outcomes	Key findings
Jiang et al. (2016) [48]	Prospective case series	174 diabetic patients without DES undergoing phacoemulsification	474 age-matched nondiabetic patients	Ocular symptom scores (OSDI), tear film stability (TBUT), corneal epithelium integrity (CFS), and tear secretion (SIT)	Diabetic patients after cataract surgery showed a higher incidence of DES (17.1% at seven days post-op) compared to nondiabetic patients (8.1%). DES in diabetic patients was reduced to 4.8% at one month and to zero at three months postoperatively. Symptoms and tear stability worsened in diabetics at seven days and one month but returned to preoperative levels by three months, slower than in nondiabetic patients. Corneal staining and tear secretion did not significantly change postoperatively in either group.
Nichols and Sinnott (2006) [49]	Cross-sectional study	Tear film analysis (interferometry, osmolality, phenol red thread, meibography, fluorescein, and lissamine green staining), contact lens analysis (water content, refractive index, and material), and patient-related factors (gender, sociodemographic, education, income, and medical health)	No specific intervention was mentioned	Self-reported contact lens-related dry eye	Female gender associated with dry eye status (P = 0.007); lenses with higher nominal water content linked to dry eye (P = 0.002); rapid pre-lens tear film thinning time associated with dry eye (P = 0.008); frequent use of over-the-counter pain medication linked to dry eye (P = 0.02); limbal injection associated with dry eye (P = 0.03); increased tear film osmolality linked to dry eye (P = 0.05).
Gong et al. (2022) [50]	Prospective, observational study	Preoperative meibomian gland status	-	Ocular surface parameters: OSDI, NIBUT, tear meniscus height, and SIT	A total of 78 patients were enrolled and divided into three groups based on meibomian gland loss grade. There are no significant baseline differences in parameters. OSDI increased in all groups at one month post-op, then decreased. Group 3 had consistently higher OSDI than Group 1 at all post-op time points (P = 0.005, 0.002, and 0.034). Group 2 had a higher OSDI at three and six months vs. Group 1 (P = 0.006 and 0.029). Average NIBUT was shorter in Group 3 since one month post-op compared to Groups 1 and 2. The grade of meibomian gland loss correlated positively with total OSDI and vision-related scores at one and three months post-op. Positive correlation with environmental score at six months post-op.
Rabina et al. (2019) [51]	Retrospective case series	PRK for myopia	Patients without dry eye disease	Postoperative pain, discomfort, photophobia, foreign body sensation, satisfaction with vision, and frequency of usage of anesthetic drops	Preoperative dry eye symptoms (OSDI score >0) were associated with more postoperative pain and discomfort. Patients with moderate to severe symptoms suffered more pain, photophobia, and epiphora, negatively impacting satisfaction with the procedure. No significant difference in postoperative subjective visual quality was observed between the groups.
Tanbakouee et al. (2016) [52]	Prospective, nonrandomized study	PRK	N/A	Schirmer test, TBUT, and OSDI questionnaire	Tear secretion decreased significantly in the low Schirmer test value group; tear stability was compromised more in the normal Schirmer test value group.
Rush et al. (2023) [53]	Prospective, observational case series	Bilateral myopic FS-LASIK	None	Patient-reported dry eye symptoms, OSI, TFO, and automated TBUTs	The Dry Eye Symptom Index score improved significantly from 2.3 (95% CI: 2.0-2.6) pretreatment to 1.3 (95% CI: 1.0-1.5) at six months post-FS-LASIK (P < 0.0001). Subset analysis showed improvement in “grittiness” (P = 0.001) but not in “light sensitivity” or “soreness” (P = 0.13 and P = 0.24, respectively). There were no significant changes in OSI, TFO, or TBUT at six months (P > 0.05 for all). No adverse events or complications occurred during the study period.
Acan and Kurtgoz (2017) [54]	Prospective cohort study	Patients (Group 1) with depression and/or anxiety disorder using selective serotonin reuptake inhibitors	Healthy volunteers (Group 2)	Corneal and conjunctival fluorescein staining, Oxford scoring, TBUT, Schirmer 1 test, and OSDI score	In Group 1, the mean TBUT was significantly lower (7.05 ± 4.86 seconds) compared to Group 2 (12.53 ± 4.75 seconds, P < 0.001). The mean superficial punctate staining (Oxford scale) was higher in Group 1 (0.78 ± 0.76) than in Group 2 (0.11 ± 0.32, P < 0.001). OSDI scores were also higher in Group 1 (32.07) compared to Group 2 (16.31, P < 0.001). Selective serotonin reuptake inhibitor usage may impact tear film stability and influence the ocular surface.
Nättinen et al. (2020) [55]	Prospective	LASIK surgery	Pre-op	Tear protein changes 1.5 hours and one-month post-LASIK using SWATH-MS	SWATH-MS identified 158 proteins with altered expression 1.5 hours post-operation, mostly linked to migration and inflammation responses, returning to baseline within a month. Identified proteins were correlated with surgical variables like amount of correction, flap thickness, and diameter. Immune cell migration and inflammation-associated changes were noted post-operation. Most proteins returned to preoperative levels within a month. Identified proteins’ potential targets for LASIK-induced biochemical process modification.
Aragona et al. (2005) [56]	Clinical study	Group 1: 0.1% indomethacin, one drop three times a day; Group 2: 0.1% diclofenac, the same regimen	No systemic NSAIDs allowed; use of tear substitute permitted	Corneal sensitivity, corneal staining, TBUT, and ocular discomfort	Both groups showed a statistically significant reduction in corneal sensitivity at day 30 (P < 0.05). The diclofenac-treated group exhibited significantly lower sensitivity compared to the indomethacin-treated group (P < 0.05). The corneal fluorescein score was significantly worse in Group 2, seven days after discontinuation of therapy (P = 0.02). The ocular discomfort score significantly reduced in both groups from day 15 (P < 0.05). NSAIDs can alleviate symptoms of ocular discomfort in SS patients, but caution and close monitoring are essential, with prompt discontinuation if corneal epithelial defects develop or worsen during treatment.
Salib et al. (2006) [57]	Randomized controlled trial	Cyclosporine 0.05% ophthalmic emulsion twice a day, starting one month before LASIK, discontinued 48 hours post-surgery, and resumed for three months	Unpreserved artificial tears used as needed	Schirmer scores, OSDI questionnaire, BCVA, superficial punctate keratitis, and refractive outcomes	The cyclosporine group showed significant increases in Schirmer scores before surgery and one week, one month, and six months after LASIK (P > 0.001).
Titiyal et al. (2023) [58]	Randomized controlled trial	Group I: standard treatment (moxifloxacin, prednisolone, and carboxymethyl cellulose)	Group I: standard treatment	Primary outcome: change in OSDI at six months; secondary outcomes: TBUT, Schirmer score, TFO, tear film MMP-9, and visual acuity	At six months, both CsA and CHQ groups showed significantly better OSDI, MMP-9, tear osmolarity, TBUT, and Schirmer scores compared to controls (P < 0.001). OSDI, tear osmolarity, TBUT, and MMP-9 levels were comparable between the CsA and CHQ groups (P > 0.05). Tear film MMP-9 levels in the CsA group at six months were comparable to baseline (P = 0.09). The Schirmer score showed no significant change from baseline in the CsA group and was significantly better than the CHQ group at six months (P = 0.02). Visual acuity was comparable in all three groups. Adverse effects were reported by ten patients (three in the CsA group and seven in the CHQ group; P = 0.28). Both CsA and CHQ are effective adjuncts to standard therapy for maintaining ocular surface stability after refractive surgery. CsA has a more potent and sustained anti-inflammatory effect, with fewer ocular irritative effects.
Hessert et al. (2013) [59]	Randomized clinical trial	Topical CsA 0.05% emulsion twice daily for three months postoperatively in PRK or LASIK patients	Standard postoperative treatment regimen without CsA	Visual acuity, mesopic contrast acuity, refractions, ocular symptoms, and tear-film samples (cytokines and chemokines)	The addition of topical CsA twice a day for three months after PRK or LASIK did not confer special benefits in achieving target refraction, final UDVA, rate of visual recovery, or patient symptoms. Tear-film composition based on measurement of inflammatory mediators (cytokines) also showed no significant difference.
Noda-Tsuruya et al. (2006) [60]	Prospective, randomized study	Autologous serum eye drops	Artificial tears	Schirmer test, TBUT, Rose Bengal, and fluorescein staining	TBUT was greater in the autologous serum group at six months post-LASIK. Lower Rose Bengal score in the autologous serum group at one and three months post-LASIK. There was no significant difference in the Schirmer test or fluorescein scores between groups. In the autologous serum group, improved TBUT, reduced Rose Bengal, and fluorescein scores post-LASIK. There was no difference in subjective dryness scores between the groups.
Mondy et al. (2015) [61]	Prospective study	Autologous serum eye drops	No specific control group mentioned	Self-reported ocular symptoms, visual-related functioning, and quality of life	Significant improvements in dryness, ocular pain, and grittiness at two and 12 months post-treatment. Patients reported feeling more in control and needing less assistance from others at 12 months.
Allegri et al. 2014 [62]	Randomized, double-blind, placebo-controlled clinical trial	0.5% indomethacin eye drops	Placebo (vehicle of indomethacin)	Visual acuity testing; slit-lamp examination; IOP evaluation; Heidelberg Spectralis optical coherence tomography CFT measurement; subjective symptoms and tolerability	There was a significant reduction in CFT (P < 0.0001) from baseline to the six-month visit in the 0.5% indomethacin-treated group. Significant improvement in VA only occurred in the 0.5% indomethacin-treated group. Global reduction of discomfort symptoms in both groups (P < 0.001).
Koh et al. (2008) [63]	Observational	Not applicable	Not applicable	HOA measurements post-blink in patients with dry eye	Dry eyes with central SPK had significantly greater total ocular HOAs than those without SPK. The sequential pattern of total ocular HOAs differed between dry eyes with and without central SPK. Increased HOAs in the dry eye might be partially due to SPK above the optical zone. Low tear volume in the dry eye might not cause sequential increases in HOAs after blinking. Sequential measurement of HOAs could be valuable for evaluating optical quality changes in dry eye patients.
Alio et al. (2017) [64]	Prospective interventional	Autologous E-PRP eye drops	None	Dry eye symptoms, CFS, CDVA improvement, and others	Dry eye symptoms improved in 85% of cases. Positive changes in corneal fluorescein staining. Conjunctival hyperemia improvement in 93.3% of patients. Significant CDVA improvement.
Albietz et al. (2004) [65]	Retrospective analysis and case study series	LASIK surgery and management of chronic dry eye	None	Regression after LASIK and myopic outcomes	Regression after LASIK was higher in patients with chronic dry eye (27% vs. 7% without) (P < 0.001).

Eligibility Screening

After removing duplicates, the reviewer executed a rigorous screening process based on titles and abstracts, followed by a meticulous assessment of full-text articles. Inclusion criteria centered on studies specifically addressing post-refractive surgery dry eye, covering pathophysiology, risk factors, and management strategies. Exclusion criteria included studies such as case-control studies, case series, case reports, and opinion reports. Any disparities during the screening process were resolved through consensus achieved via reviewer discussion.

Data Extraction

**Figure 1:**
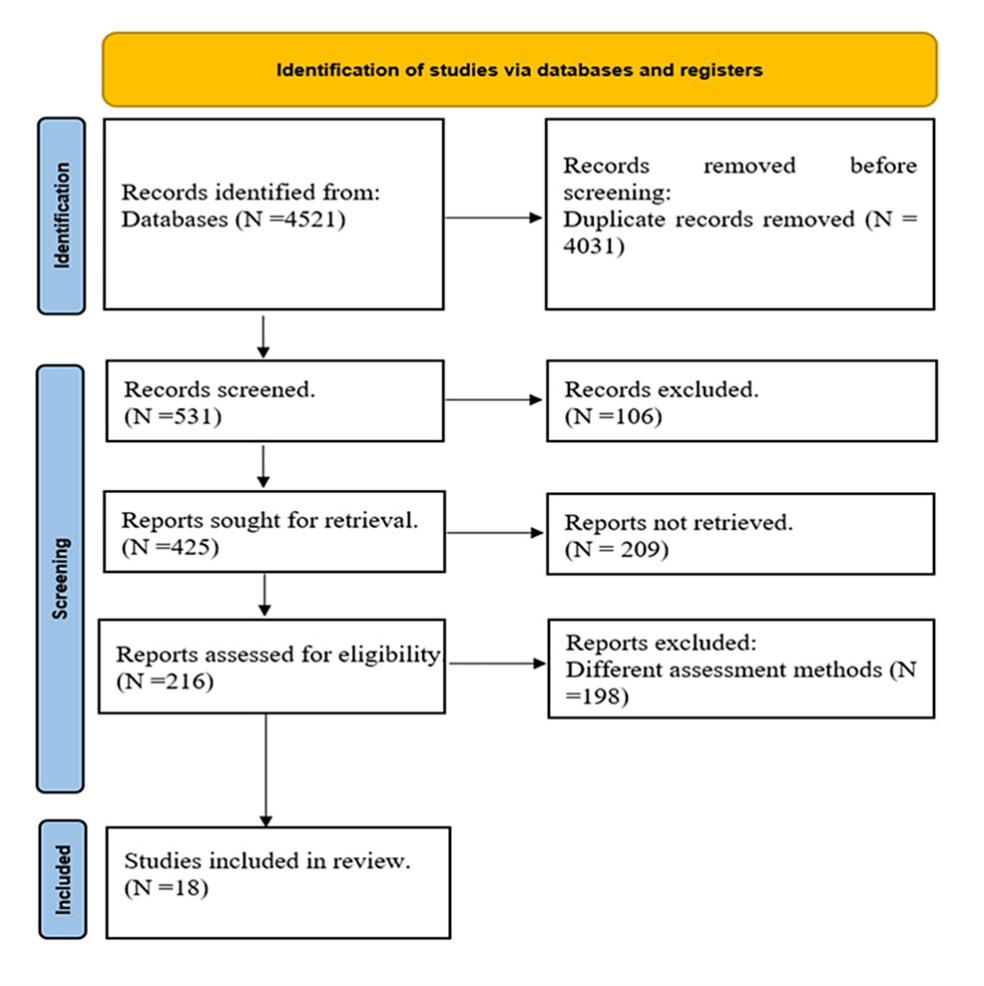
PRISMA flow diagram PRISMA, Preferred Reporting Items for Systematic reviews and Meta-Analyses

The first database search turned up a total of 4,521 documents. After duplicates were removed, 531 articles were evaluated based just on their title and abstract; 106 of them were rejected. Reports assessed the remaining 425 papers’ eligibility (18 were selected in the end for the full-text review out of a total of 216) [48,49,50-65]. Figure 1 explains the PRISMA flow diagram.

Quality Assessment

The reviewer conducted a thorough evaluation of the methodological quality and risk of bias of all eligible studies. We evaluated all studies as an independent observational cohort, by using a modified version of ROBVIS2, which was developed during the Evidence Synthesis Hackathon. This web app is built on the ROBVIS R package [66]. Discrepancies in the assessment were resolved through consensus.

Data Analysis

This systematic review will employ narrative synthesis and thematic analysis:

Narrative synthesis: This qualitative methodology involves summarizing and interpreting findings from selected studies pertaining to the management of post-refractive surgery dry eye. It aims to provide a descriptive and critical synthesis, highlighting implications for healthcare practitioners and patients alike.

Thematic analysis: Utilizing thematic analysis, common themes, patterns, and implications observed across selected studies will be identified and categorized. Findings related to pathophysiology, risk factors, and management approaches for post-refractive surgery dry eye will be coded, enabling a deeper exploration of connections and variations within these themes. This analysis aims to offer a comprehensive understanding of the effectiveness and challenges associated with managing post-refractive surgery dry eye.

Results

The Quality Assessment

**Figure 2:**
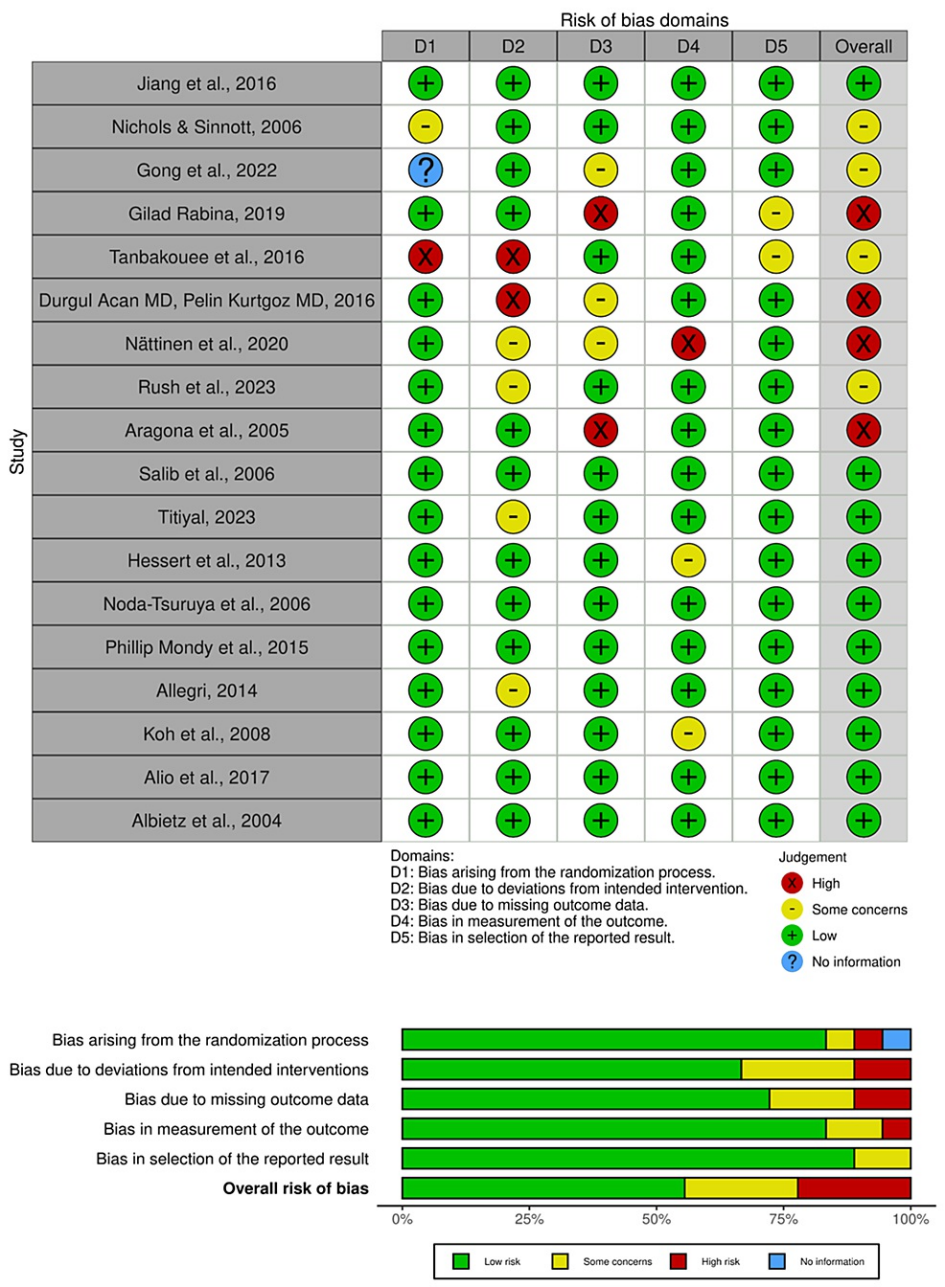
Summary of risk of bias

The risk of bias assessment across the selected studies presents varying levels of concern across distinct domains (Figure 2). Jiang et al. (2016) demonstrated consistently low bias across all aspects assessed, indicating a robust methodology and minimizing the potential for bias [48]. Nichols and Sinnott (2006) showed mostly low bias across domains, yet some concerns arose, potentially impacting the reported results [49]. Gong et al. (2022) lacked information on the bias due to the randomization process but overall demonstrated low concerns in other areas, with a few aspects raising some concern regarding reported outcomes [50].

However, Rabina et al. (2019) and Tanbakouee et al. (2016) exhibited deeper concerns, particularly in areas of missing outcome data and deviation from intended interventions, potentially impacting the reliability of their findings [51,52]. Similarly, Acan and Kurtgoz (2017), Nättinen et al. (2020), and Aragona et al. (2005) displayed higher concerns in multiple domains, including measurement of outcomes and overall reported results, suggesting a greater potential for bias influencing their conclusions [54-56].

Conversely, studies like Rush et al. (2023), Titiyal et al. (2023), and Allegri et al. (2014) demonstrated generally low concerns across most domains, indicating a more robust methodology and reduced potential for bias [53,58,62]. Yet, a few areas raised some concerns in certain studies, such as Hessert et al. (2013), which displayed some concerns regarding the measurement of outcomes [59]. Similarly, studies like Noda-Tsuruya et al. (2006) and Koh et al. (2008), while generally low in bias, showed some concerns related to the selection of reported results, hinting at potential selective reporting bias [60,63].

Overall, the studies encompass a spectrum of bias concerns across various domains. While some studies exhibited robust methodologies with minimal potential for bias, others revealed multiple areas of concern, particularly in missing outcome data, deviation from intended interventions, and selection of reported results, emphasizing the need for cautious interpretation of their findings.

Main Outcomes

Understanding the intricate dynamics of dry eye syndrome (DES) and its multifaceted outcomes following various interventions and surgical procedures is a critical pursuit in contemporary ophthalmology. Table 1 presents a comprehensive overview of studies focusing on the pathophysiology, interventions, and management approaches related to DES across diverse clinical settings. These studies, ranging from prospective clinical trials to retrospective analyses and case series, collectively shed light on several pivotal facets of DES, including its postoperative manifestations, the impact of specific interventions, associations with risk factors, and the influence of underlying conditions. Based on the data extracted from the 18 selected articles (Table 1), the following outcomes were extracted:

Studies such as Jiang et al. (2016), Rabina et al. (2019), and Rush et al. (2023) delve into the alterations in dry eye symptoms post-ocular surgeries [48,51,53]. Jiang et al. (2016) observed a higher incidence of DES in diabetic patients after cataract surgery, noting initial exacerbation of symptoms and tear stability, which eventually returned to preoperative levels but at a slower rate compared to nondiabetic patients [48]. Rabina et al. (2019) highlighted a correlation between preoperative dry eye symptoms and increased postoperative discomfort, adversely impacting patient satisfaction [51]. Rush et al. (2023) noted improvements in dry eye symptom scores post-laser refractive surgery, specifically in symptoms related to “grittiness,” highlighting the evolving nature of postoperative dry eye symptoms [53].

Studies such as Nättinen et al. (2020), Salib et al. (2006), and Allegri et al. (2014) investigated interventions’ effects, such as LASIK surgery, topical treatments (cyclosporine and non-steroidal anti-inflammatory drugs (NSAIDs)), and autologous serum eye drops, on dry eye parameters [55,57,62]. Nättinen et al. (2020) employed proteomic analysis to identify proteins affected by LASIK, potentially serving as targets for modifying LASIK-induced biochemical processes [55]. Salib et al. (2006) reported increased tear production with cyclosporine treatment post-LASIK [57], while Allegri et al. (2014) noted reductions in discomfort symptoms with indomethacin eye drops, showcasing the potential for specific interventions to impact dry eye symptoms [62].

Associations Between Dry Eye and Risk Factors

Nichols and Sinnott (2006) and Tanbakouee et al. (2016) explored associations between DES and various risk factors [49,52]. Nichols and Sinnott (2006) linked factors like gender, contact lens properties, tear film dynamics, and medication usage to the prevalence of dry eye [49]. Tanbakouee et al. (2016) reported significant reductions in tear secretion and stability after refractive surgery, highlighting the importance of tear film quality and its impact on dry eye symptoms in different subgroups [52].

Acan and Kurtgoz (2017) and Titiyal et al. (2023) investigated the influence of underlying conditions on dry eye parameters [54,58]. Acan and Kurtgoz (2017) emphasized altered tear film stability and ocular surface changes in patients with depression and/or anxiety disorders using selective serotonin reuptake inhibitors [54]. Titiyal et al. (2023) studied the effects of adjunct treatments (CsA and CHQ) on dry eye post-refractive surgery, demonstrating their effectiveness in maintaining ocular surface stability [58].

Discussion

This systematic review offers valuable insights into the intricate pathophysiology, risk factors, and management approaches for dry eye disease, which is emerging as a frequent complication after corneal refractive procedures. The current evidence, encompassing 18 studies, highlights the complex interplay between structural damage, neurotrophic changes, and inflammation in the pathogenesis of post-refractive surgery dry eye. Multiple systemic and ocular characteristics also predispose patients to exacerbated dryness after LASIK and surface ablation techniques. While conventional therapies provide symptomatic relief, novel targeted treatments promise enhanced outcomes by promoting nerve regeneration and anti-inflammation.

Structural and Neurotrophic Changes Underlying Pathogenesis

Multiple included studies highlight the pivotal roles of neuronal and epithelial damage in the development of dry eye disease following corneal refractive procedures. Surgical transection of sub-basal nerves during stromal ablation leads to reduced corneal sensation and impaired neural reflex arcs regulating tear production and eyelid function [58,67]. Corresponding corneal hypoesthesia likely diminishes homeostatic interactions between sensory afferents and the lacrimal functional unit [68].

The intricate interplay between neuronal and epithelial changes post-corneal refractive surgeries is crucial in understanding the development of DES [69]. Studies consistently underscore the profound impact of surgical procedures on both the sensory nerves and the corneal epithelium, illuminating the multifaceted pathogenesis of this postoperative complication [70]. The surgical process, particularly during stromal ablation, inadvertently severs sub-basal nerves, causing a substantial decline in corneal sensation [16,71]. These sub-basal nerves are integral components of the corneal innervation responsible for transmitting sensory signals essential for regulating tear production and the blink reflex [72]. Disruption of these neural reflex arcs compromises the delicate equilibrium between sensory inputs and the lacrimal functional unit [72]. The resultant corneal hypoesthesia hampers the neural feedback mechanisms that normally govern tear secretion and ocular surface maintenance [13].

Surgical trauma not only affects the nerves but also impairs the corneal epithelium [73]. Limbal stem cell deficiency, triggered by surgical trauma, significantly impacts the epithelial renewal capacity [74]. This deficiency leads to impaired epithelial regeneration and repair mechanisms, contributing to epithelial thinning, irregularity, and inflammatory responses [75]. Basement membrane disruptions further exacerbate these effects, compromising the structural integrity of the corneal epithelium [76]. Ultrastructural analyses have demonstrated the breakdown of vital cell junction complexes responsible for corneal tissue stability, exacerbating the vulnerability of the ocular surface [77].

Risk Factors and Patient Selection

Among the prominent risk factors, female gender surfaces as a consistent predictor of heightened susceptibility to dry eye post-surgery. Studies underscore this finding, aligning with clinical observations that women are more prone to dry eye disorders [48]. Additionally, underlying conditions such as thyroid eye disease, meibomian gland dysfunction, and preexisting dry eye substantially contribute to the risk profile. The presence of these conditions amplifies the vulnerability of patients to postoperative dry eye complications [78].

The choice of surgical technique plays a pivotal role in dictating the incidence and severity of dry eye following refractive surgeries [79]. Higher ablation depths, often associated with certain procedures like LASIK, pose an increased risk. Studies elucidate that the use of LASIK over surface ablation techniques amplifies the likelihood of postoperative dry eye symptoms [80]. Moreover, corneal characteristics such as flattened corneas and lower tear meniscus heights serve as additional risk indicators, contributing to the overall predisposition of patients to develop dry eye post-surgery [81].

This comprehensive identification of preoperative risk factors offers crucial insights into patient selection for refractive surgeries. Understanding and acknowledging these risk determinants is paramount in clinical decision-making, aiding ophthalmologists in evaluating patient candidacy for these procedures [82]. For borderline candidates, this knowledge allows for a nuanced approach, focusing on the optimization of modifiable risk factors before proceeding with surgery. In cases where refractive surgery remains a viable option, customized ablation profiles and the consideration of alternative techniques, such as small incision lenticule extraction, prove beneficial [83]. These strategies aim to mitigate the risk of corneal nerve damage and minimize the likelihood of developing postoperative dry eye, ultimately enhancing patient outcomes and satisfaction.

Anti-inflammatory and Reparative Therapies Show Promise

The emergence of novel therapeutic approaches targeting the inflammatory pathways implicated in DES post-corneal refractive surgeries represents a promising avenue for managing this challenging condition [84]. Conventional lubricants like artificial tears, gels, and ointments have been used as a first step in providing temporary comfort by enhancing the moisture of the surface of the eye [85]. 

Studies have highlighted the potential of anti-inflammatory agents, such as corticosteroids, NSAIDs, cyclosporine, and lifitegrast, in mitigating the inflammatory processes involved in dry eye [86]. These treatments aim to modulate the immune response and reduce ocular surface inflammation, thereby alleviating symptoms and improving tear film stability. Yet, despite their efficacy, concerns persist regarding the long-term use of these medications due to potential adverse effects [87].

In parallel, innovative modalities have emerged, showing early promise in addressing the multifactorial nature of DES [1]. Autologous serum, containing growth factors and anti-inflammatory cytokines, has shown potential for restoring ocular surface integrity by promoting epithelial healing and reducing inflammation [88]. Moreover, therapies targeting NGFs exhibit significant potential for directly stimulating corneal epithelial regeneration and promoting sensory nerve regrowth, thereby aiding in tear production and ocular surface stability [89].

Despite the promising initial findings, further robust clinical trials are essential to ascertain the optimal dosing, long-term safety, and overall efficacy of these emerging therapies. The intricate balance between therapeutic efficacy and potential adverse effects necessitates thorough investigation and validation of these novel modalities before their widespread clinical application [90]. Achieving a deeper understanding of their mechanisms of action and conducting extensive longitudinal studies will be crucial in establishing their role as viable alternatives or complementary treatments in managing DES following corneal refractive surgeries [5,91,92].

Emergence of Personalized Medicine Approaches

Targeting the inflammatory pathways associated with DES following corneal refractive procedures offers a practical approach to managing this challenging condition [93]. Conventional lubricants like artificial tears, gels, and ointments have been used as a first-line treatment to provide temporary comfort by increasing the moisture of the surface of the eye [94]. Nevertheless, the limits of their ability to treat the fundamental inflammatory processes have prompted the investigation of more focused approaches to reduce inflammation [34,92]. Research has shown that anti-inflammatory substances, including corticosteroids, NSAIDs, cyclosporine, and lifitegrast, have the ability to reduce the inflammation associated with dry eye [95]. The objective of these therapies is to regulate the immune response and decrease inflammation on the surface of the eye, with the goal of relieving symptoms and enhancing the integrity of the tear film. However, even if these drugs are effective, there are still worries about their long-term usage because of possible harmful effects [96]. Simultaneously, novel approaches have arisen that show initial potential in tackling the complex and multifaceted characteristics of DES [97]. Autologous serum, which contains growth factors and anti-inflammatory cytokines, has shown promise in repairing the ocular surface by stimulating the mending of epithelial cells and decreasing inflammation [98]. Furthermore, treatments that focus on NGFs have a considerable capacity to directly stimulate the renewal of the corneal epithelium and promote the regrowth of sensory nerves. This, in turn, helps in the production of tears and the stability of the ocular surface [99]. Furthermore, studies investigating the use of MMPIs indicate a possible approach to mitigate the breakdown of extracellular matrix, therefore improving the attachment and strength of epithelial cells [100]. These inhibitors show potential in maintaining the structural integrity of the ocular surface, which is essential in controlling the advancement of DES.

## Conclusions

This systematic review, integrating 18 studies, provides significant insights into the pathophysiological mechanisms, risk factors, and therapeutic approaches for managing dry eye disease as a common complication after corneal refractive surgeries. Surgical disruption of corneal nerves and architecture triggers neurotrophic epitheliopathy, apoptosis, inflammation, and lacrimal dysfunction, culminating in ocular discomfort, visual disturbances, and tear film instability. Multiple systemic and ocular factors predispose patients to clinically significant postoperative dry eye that impairs quality of life. Female gender, thyroid eye disease, meibomian gland dysfunction, younger ages, preexisting dry eye, higher attempted correction, deeper ablations, and use of LASIK over surface techniques are key risks warranting optimization before proceeding with elective surgery. Schirmer’s scores under 10 mm and tear film breakup times below 10 seconds should trigger caution. Chronic inflammation from autoimmune conditions is an absolute contraindication needing resolution preoperatively.

## References

[1] Buckley RJ (2018). Assessment and management of dry eye disease. Eye (Lond).

[2] Sahay P, Bafna RK, Reddy JC, Vajpayee RB, Sharma N (2021). Complications of laser-assisted in situ keratomileusis. Indian J Ophthalmol.

[3] Nair S, Kaur M, Sharma N, Titiyal JS (2023). Refractive surgery and dry eye - an update. Indian J Ophthalmol.

[4] Shtein RM (2011). Post-LASIK dry eye. Expert Rev Ophthalmol.

[5] Tamimi A, Sheikhzadeh F, Ezabadi SG (2023). Post-LASIK dry eye disease: a comprehensive review of management and current treatment options. Front Med (Lausanne).

[6] Kojima T, Dogru M, Kawashima M, Nakamura S, Tsubota K (2020). Advances in the diagnosis and treatment of dry eye. Prog Retin Eye Res.

[7] Vereertbrugghen A, Galletti JG (2022). Corneal nerves and their role in dry eye pathophysiology. Exp Eye Res.

[8] Bandeira F, Yusoff NZ, Yam GH, Mehta JS (2019). Corneal re-innervation following refractive surgery treatments. Neural Regen Res.

[9] Raevdal P, Grauslund J, Vestergaard AH (2019). Comparison of corneal biomechanical changes after refractive surgery by noncontact tonometry: small-incision lenticule extraction versus flap-based refractive surgery - a systematic review. Acta Ophthalmol.

[10] Zhang X, M VJ, Qu Y (2017). Dry eye management: targeting the ocular surface microenvironment. Int J Mol Sci.

[11] Nettune GR, Pflugfelder SC (2010). Post-LASIK tear dysfunction and dysesthesia. Ocul Surf.

[12] Foulks GN (2010). Tear dysfunction from lacrimal gland to LASIK. Ocul Surf.

[13] Labetoulle M, Baudouin C, Calonge M (2019). Role of corneal nerves in ocular surface homeostasis and disease. Acta Ophthalmol.

[14] Nurković JS, Vojinović R, Dolićanin Z (2020). Corneal stem cells as a source of regenerative cell-based therapy. Stem Cells Int.

[15] Sejpal K, Bakhtiari P, Deng SX (2013). Presentation, diagnosis and management of limbal stem cell deficiency. Middle East Afr J Ophthalmol.

[16] Tomás-Juan J, Murueta-Goyena Larrañaga A, Hanneken L (2015). Corneal regeneration after photorefractive keratectomy: a review. J Optom.

[17] Soiberman U, Foster JW, Jun AS, Chakravarti S (2017). Pathophysiology of keratoconus: what do we know today. Open Ophthalmol J.

[18] Efraim Y, Chen FY, Stashko C, Cheong KN, Gaylord E, McNamara N, Knox SM (2020). Alterations in corneal biomechanics underlie early stages of autoimmune-mediated dry eye disease. J Autoimmun.

[19] (2007). The definition and classification of dry eye disease: report of the Definition and Classification Subcommittee of the International Dry Eye Workshop (2007). Ocul Surf.

[20] Solomon R, Donnenfeld ED, Perry HD (2004). The effects of LASIK on the ocular surface. Ocul Surf.

[21] Shehadeh-Mashor R, Mimouni M, Shapira Y, Sela T, Munzer G, Kaiserman I (2019). Risk factors for dry eye after refractive surgery. Cornea.

[22] Tripathi R, Giuliano EA, Gafen HB (2019). Is sex a biological variable in corneal wound healing?. Exp Eye Res.

[23] Horng HC, Chang WH, Yeh CC (2017). Estrogen effects on wound healing. Int J Mol Sci.

[24] Sheppard JD, Nichols KK (2023). Dry eye disease associated with meibomian gland dysfunction: focus on tear film characteristics and the therapeutic landscape. Ophthalmol Ther.

[25] Erie JC, McLaren JW, Hodge DO, Bourne WM (2005). Long-term corneal keratoctye deficits after photorefractive keratectomy and laser in situ keratomileusis. Trans Am Ophthalmol Soc.

[26] Gjerdrum B, Gundersen KG, Lundmark PO, Potvin R, Aakre BM (2020). Prevalence of signs and symptoms of dry eye disease 5 to 15 after refractive surgery. Clin Ophthalmol.

[27] Sánchez-Brau M, Seguí-Crespo M, Cantó-Sancho N, Tauste A, Ramada JM (2023). What are the dry eye questionnaires available in the scientific literature used for? A scoping review. Am J Ophthalmol.

[28] Ngo W, Situ P, Keir N, Korb D, Blackie C, Simpson T (2013). Psychometric properties and validation of the Standard Patient Evaluation of Eye Dryness questionnaire. Cornea.

[29] Asiedu K, Kyei S, Mensah SN, Ocansey S, Abu LS, Kyere EA (2016). Ocular Surface Disease Index (OSDI) versus the Standard Patient Evaluation of Eye Dryness (SPEED): a study of a nonclinical sample. Cornea.

[30] Pucker AD, Dougherty BE, Jones-Jordan LA, Kwan JT, Kunnen CM, Srinivasan S (2018). Psychometric analysis of the SPEED Questionnaire and CLDEQ-8. Invest Ophthalmol Vis Sci.

[31] Chuang J, Shih KC, Chan TC, Wan KH, Jhanji V, Tong L (2017). Preoperative optimization of ocular surface disease before cataract surgery. J Cataract Refract Surg.

[32] D'Souza S, James E, Swarup R, Mahuvakar S, Pradhan A, Gupta K (2020). Algorithmic approach to diagnosis and management of post-refractive surgery dry eye disease. Indian J Ophthalmol.

[33] Özbek-Uzman S, Yalnız-Akkaya Z, Şingar Özdemir E, Burcu A (2022). Treatment of persistent epithelial defects with single-dose autologous serum eye drops. Eur J Ophthalmol.

[34] Weng J, Fink MK, Sharma A (2023). A critical appraisal of the physicochemical properties and biological effects of artificial tear ingredients and formulations. Int J Mol Sci.

[35] Donthineni PR, Deshmukh R, Ramamurthy C, Sangwan VS, Mehta JS, Basu S (2023). Management of cataract in dry eye disease: preferred practice pattern guidelines. Indian J Ophthalmol.

[36] Qiao J, Yan X (2013). Emerging treatment options for meibomian gland dysfunction. Clin Ophthalmol.

[37] Singla S, Sarkar L, Joshi M (2019). Comparison of topical cyclosporine alone and topical loteprednol with cyclosporine in moderate dry eye in Indian population: a prospective study. Taiwan J Ophthalmol.

[38] Marcum ZA, Hanlon JT (2010). Recognizing the risks of chronic nonsteroidal anti-inflammatory drug use in older adults. Ann Longterm Care.

[39] Blanco-Mezquita T, Martinez-Garcia C, Proença R, Zieske JD, Bonini S, Lambiase A, Merayo-Lloves J (2013). Nerve growth factor promotes corneal epithelial migration by enhancing expression of matrix metalloprotease-9. Invest Ophthalmol Vis Sci.

[40] Shiju TM, Sampaio LP, Hilgert GS, Wilson SE (2023). Corneal epithelial basement membrane assembly is mediated by epithelial cells in coordination with corneal fibroblasts during wound healing. Mol Vis.

[41] Geerling G, Maclennan S, Hartwig D (2004). Autologous serum eye drops for ocular surface disorders. Br J Ophthalmol.

[42] Martinez JD, Arrieta E, Naranjo A (2021). Rose bengal photodynamic antimicrobial therapy: a pilot safety study. Cornea.

[43] Huang R, Su C, Fang L, Lu J, Chen J, Ding Y (2022). Dry eye syndrome: comprehensive etiologies and recent clinical trials. Int Ophthalmol.

[44] Miura M, Inomata T, Nakamura M (2022). Prevalence and characteristics of dry eye disease after cataract surgery: a systematic review and meta-analysis. Ophthalmol Ther.

[45] Aragona P, Giannaccare G, Mencucci R, Rubino P, Cantera E, Rolando M (2021). Modern approach to the treatment of dry eye, a complex multifactorial disease: a P.I.C.A.S.S.O. board review. Br J Ophthalmol.

[46] Page MJ, McKenzie JE, Bossuyt PM (2021). The PRISMA 2020 statement: an updated guideline for reporting systematic reviews. BMJ.

[47] Shamseer L, Moher D, Clarke M (2015). Preferred reporting items for systematic review and meta-analysis protocols (PRISMA-P) 2015: elaboration and explanation. BMJ.

[48] Jiang D, Xiao X, Fu T, Mashaghi A, Liu Q, Hong J (2016). Transient tear film dysfunction after cataract surgery in diabetic patients. PLoS ONE.

[49] Nichols JJ, Sinnott LT (2006). Tear film, contact lens, and patient-related factors associated with contact lens-related dry eye. Invest Ophthalmol Vis Sci.

[50] Gong Q, Li A, Chen L (2022). Evaluation of dry eye after refractive surgery according to preoperative meibomian gland status. Front Med (Lausanne).

[51] Rabina G, Boguslavsky II, Mimouni M, Kaiserman I (2019). The association between preoperative dry eye symptoms and postoperative discomfort in patients underwent photorefractive keratectomy. J Ophthalmol.

[52] Tanbakouee E, Ghoreishi M, Aghazadeh-Amiri M, Tabatabaee M, Mohammadinia M (2016). Photorefractive keratectomy for patients with preoperative low Schirmer test value. J Curr Ophthalmol.

[53] Rush S, Pickett CJ, Rush RB (2023). Patient-reported dry eye outcomes after myopic femtosecond-LASIK: a 6-month prospective analysis. Clin Ophthalmol.

[54] Acan D, Kurtgoz P (2017). Influence of selective serotonin reuptake inhibitors on ocular surface. Clin Exp Optom.

[55] Nättinen J, Mäkinen P, Aapola U, Orsila L, Pietilä J, Uusitalo H (2020). Early changes in tear film protein profiles after femtosecond LASIK surgery. Clin Proteomics.

[56] Aragona P, Stilo A, Ferreri F, Mobrici M (2005). Effects of the topical treatment with NSAIDs on corneal sensitivity and ocular surface of Sjögren's syndrome patients. Eye (Lond).

[57] Salib GM, McDonald MB, Smolek M (2006). Safety and efficacy of cyclosporine 0.05% drops versus unpreserved artificial tears in dry-eye patients having laser in situ keratomileusis. J Cataract Refract Surg.

[58] Titiyal JS, Goswami A, Kaur M, Sharma N, Maharana PK, Velpandian T, Pandey RM (2023). Impact of topical cyclosporine-a or topical chloroquine on post-LASIK ocular surface stability - a randomized controlled trial. Curr Eye Res.

[59] Hessert D, Tanzer D, Brunstetter T, Kaupp S, Murdoch D, Mirzaoff M (2013). Topical cyclosporine A for postoperative photorefractive keratectomy and laser in situ keratomileusis. J Cataract Refract Surg.

[60] Noda-Tsuruya T, Asano-Kato N, Toda I, Tsubota K (2006). Autologous serum eye drops for dry eye after LASIK. J Refract Surg.

[61] Mondy P, Brama T, Fisher J, Gemelli CN, Chee K, Keegan A, Waller D (2015). Sustained benefits of autologous serum eye drops on self-reported ocular symptoms and vision-related quality of life in Australian patients with dry eye and corneal epithelial defects. Transfus Apher Sci.

[62] Allegri P, Murialdo U, Peri S (2014). Randomized, double-blind, placebo-controlled clinical trial on the efficacy of 0.5% indomethacin eye drops in uveitic macular edema. Invest Ophthalmol Vis Sci.

[63] Koh S, Maeda N, Hirohara Y (2008). Serial measurements of higher-order aberrations after blinking in patients with dry eye. Invest Ophthalmol Vis Sci.

[64] Alio JL, Rodriguez AE, Abdelghany AA, Oliveira RF (2017). Autologous platelet-rich plasma eye drops for the treatment of post-LASIK chronic ocular surface syndrome. J Ophthalmol.

[65] Albietz JM, Lenton LM, McLennan SG (2004). Chronic dry eye and regression after laser in situ keratomileusis for myopia. J Cataract Refract Surg.

[66] McGuinness LA, Higgins JP (2021). Risk-of-bias VISualization (robvis): an R package and Shiny web app for visualizing risk-of-bias assessments. Res Synth Methods.

[67] Bech F, González-González O, Artime E (2018). Functional and morphologic alterations in mechanical, polymodal, and cold sensory nerve fibers of the cornea following photorefractive keratectomy. Invest Ophthalmol Vis Sci.

[68] Shaheen BS, Bakir M, Jain S (2014). Corneal nerves in health and disease. Surv Ophthalmol.

[69] Yang LW, Mehta JS, Liu YC (2021). Corneal neuromediator profiles following laser refractive surgery. Neural Regen Res.

[70] Sepulveda-Beltran PA, Levine H, Chang VS, Gibbons A, Martinez JD (2022). Complications in retinal surgery: a review of corneal changes following vitreoretinal procedures. Int Ophthalmol Clin.

[71] Calvillo MP, McLaren JW, Hodge DO, Bourne WM (2004). Corneal reinnervation after LASIK: prospective 3-year longitudinal study. Invest Ophthalmol Vis Sci.

[72] Dragnea DC, Krolo I, Koppen C, Faris C, Van den Bogerd B, Ní Dhubhghaill S (2023). Corneal neurotization-indications, surgical techniques and outcomes. J Clin Med.

[73] Yang AY, Chow J, Liu J (2018). Corneal innervation and sensation: the eye and beyond. Yale J Biol Med.

[74] Notara M, Lentzsch A, Coroneo M, Cursiefen C (2018). The role of limbal epithelial stem cells in regulating corneal (lymph)angiogenic privilege and the micromilieu of the limbal niche following UV exposure. Stem Cells Int.

[75] Li Y, Rao X, Tang P (2021). Bach2 deficiency promotes intestinal epithelial regeneration by accelerating DNA repair in intestinal stem cells. Stem Cell Reports.

[76] Torricelli AA, Singh V, Santhiago MR, Wilson SE (2013). The corneal epithelial basement membrane: structure, function, and disease. Invest Ophthalmol Vis Sci.

[77] Mort RL, Douvaras P, Morley SD, Dorà N, Hill RE, Collinson JM, West JD (2012). Stem cells and corneal epithelial maintenance: insights from the mouse and other animal models. Results Probl Cell Differ.

[78] Inoue S, Kawashima M, Arita R, Kozaki A, Tsubota K (2020). Investigation of meibomian gland function and dry eye disease in patients with Graves' ophthalmopathy. J Clin Med.

[79] Denoyer A, Landman E, Trinh L, Faure JF, Auclin F, Baudouin C (2015). Dry eye disease after refractive surgery: comparative outcomes of small incision lenticule extraction versus LASIK. Ophthalmology.

[80] Moshirfar M, Wang Q, Theis J, Porter KC, Stoakes IM, Payne CJ, Hoopes PC (2023). Management of corneal haze after photorefractive keratectomy. Ophthalmol Ther.

[81] Tung CI, Perin AF, Gumus K, Pflugfelder SC (2014). Tear meniscus dimensions in tear dysfunction and their correlation with clinical parameters. Am J Ophthalmol.

[82] Nuliqiman M, Xu M, Sun Y (2023). Artificial intelligence in ophthalmic surgery: current applications and expectations. Clin Ophthalmol.

[83] Moshirfar M, Shah TJ, Masud M, Fanning T, Linn SH, Ronquillo Y, Hoopes PC (2018). A modified small-incision lenticule intrastromal keratoplasty (sLIKE) for the correction of high hyperopia: a description of a new surgical technique and comparison to lenticule intrastromal keratoplasty (LIKE). Med Hypothesis Discov Innov Ophthalmol.

[84] Shaban MM, Sharaa HM, Amer FG, Shaban M (2024). Effect of digital based nursing intervention on knowledge of self-care behaviors and self-efficacy of adult clients with diabetes. BMC Nurs.

[85] O'Neil EC, Henderson M, Massaro-Giordano M, Bunya VY (2019). Advances in dry eye disease treatment. Curr Opin Ophthalmol.

[86] Srinivasan S, Williams R (2022). Propylene glycol and hydroxypropyl guar nanoemulsion - safe and effective lubricant eye drops in the management of dry eye disease. Clin Ophthalmol.

[87] Zhuang D, Misra SL, Mugisho OO, Rupenthal ID, Craig JP (2023). NLRP3 inflammasome as a potential therapeutic target in dry eye disease. Int J Mol Sci.

[88] Rolando M, Barabino S, Giannaccare G, Aragona P (2023). Dealing with the persistent pathogenic issues of dry eye disease: the importance of external and internal stimuli and tissue responses. J Clin Med.

[89] Anitua E, Muruzabal F, Tayebba A, Riestra A, Perez VL, Merayo-Lloves J, Orive G (2015). Autologous serum and plasma rich in growth factors in ophthalmology: preclinical and clinical studies. Acta Ophthalmol.

[90] Kanu LN, Ciolino JB (2021). Nerve growth factor as an ocular therapy: applications, challenges, and future directions. Semin Ophthalmol.

[91] Sun D, Gao W, Hu H, Zhou S (2022). Why 90% of clinical drug development fails and how to improve it?. Acta Pharm Sin B.

[92] Donthineni PR, Shanbhag SS, Basu S (2021). An evidence-based strategic approach to prevention and treatment of dry eye disease, a modern global epidemic. Healthcare (Basel).

[93] Messmer EM (2022). Pathophysiology of dry eye disease and novel therapeutic targets. Exp Eye Res.

[94] Doughty MJ, Glavin S (2009). Efficacy of different dry eye treatments with artificial tears or ocular lubricants: a systematic review. Ophthalmic Physiol Opt.

[95] Nguyen A, Kolluru A, Beglarian T (2023). Dry eye disease: a review of anti-inflammatory therapies. Taiwan J Ophthalmol.

[96] Colligris B, Alkozi HA, Pintor J (2014). Recent developments on dry eye disease treatment compounds. Saudi J Ophthalmol.

[97] Conrady CD, Joos ZP, Patel BC (2016). Review: the lacrimal gland and its role in dry eye. J Ophthalmol.

[98] Pınarlı FA, Okten G, Beden U (2014). Keratinocyte growth factor-2 and autologous serum potentiate the regenerative effect of mesenchymal stem cells in cornea damage in rats. Int J Ophthalmol.

[99] Bakhsh E, Alkhaldi M, Shaban M (2023). Exploring the link between maternal hematological disorders during pregnancy and neurological development in newborns: mixed cohort study. Life (Basel).

[100] Cabral-Pacheco GA, Garza-Veloz I, Castruita-De la Rosa C (2020). The roles of matrix metalloproteinases and their inhibitors in human diseases. Int J Mol Sci.

